# Aconitum Napellus

**Published:** 1852-03

**Authors:** John A. Preston

**Affiliations:** Hartland, Wisconsin


ARTICLE IX.

*Aconitum Napellus.* By John A. Preston, M. D., of Hartland, Wisconsin.

My attention was first called to this medicine by Dr. Cook, of Laghartor, L. I., formerly House Surgeon in the N. Y. city Hospital. Subsequently I was made more freely acquainted with its merits by an article published in Rankings' Abstract Vol. 1, No. 2, pp. 344, 346, to which I would refer your readers for a more extended notice. A description of the several varieties of the plant will be found in the U. S. Dispensatory. There is but one species indigenous in this country, the A. Uncinatum, named from the hook-shaped extremities of its palmato leaves, and readily recognised by its beautiful violet-hued hooded blossom. The green leaves of this species are very acrimonious, leaving upon the tongue and fauces when chewed, the stinging sensation peculiar to the other species, and closely resembling the effects of the Arum Americana. If the root of an indigenous species should prove upon trial to be as acrid as the leaves, I doubt not it might be found an efficacious and economical substitute for the imported article. The A. Napellus is undoubtedly one of the most powerful and reliable of the acro-narcotics. Its action upon the nervous system in medicinal doses is that of a direct sedative. It differs from Opium and its preparations, in being more concentrated and uniform in its action. Another advantage which it possesses over that drug is its freedom from any tendency to unduely excite the nervous system, or to produce constipation. As my experience has been entirely confined to Fleming's Tincture of the root, I shall not advert to the officinal preparations.

Dr. Fleming, the author of the paper above alluded to, prefers the tincture from its greater uniformity of operation. Its sedative action upon the heart and arteries would point to its use as a powerful antiphlogistic in fevers, and a large class of inflammatory diseases. It has not, however, come into general use as an antifebrile among regular practitioners. It is, I apprehend, better known, and more generally appreciated by the disciples of Hahunemar. His Mother Tincture, as it is called, is prepared with equal parts of the fresh juice of the root and alcohol, a preparation which probably very closely resembles Fleming's Tincture. This potent remedy, I had good means of knowing, they use in such doses as would greatly scandalize their illustrious master. It was the more particular purpose of the writer in this brief article, to call the attention of the profession to the use of the Aconitum as an anti-neuralgic. Neuralgia in its various forms has been not less the *opprobrium medicorum* than rheumatism with which it is so closely allied. To say that we have a specific for this disease in the Aconite. might savor too much of enthusiasm or dogmatism, yet I will venture the assertion that the cases of pure neuralgia which will not yield to its use, will be exceptions few and far between. I have now used it for five years, during which time I have repeatedly exhibited it in various neuralgias, and other neuropathic diseases, and have yet to find a case which it will not cure. I have often prescribed it in Rheumatism, and have rarely failed to obtain good results I have witnessed the happiest effects from its long continued use in neuralgia of the heart, after other approved remedies had signally failed. I have recently prescribed it for a gentleman, who, for three years occasionally suffered from excruciating pain, which he referred to the back part of the head and neck. Various remedies had been prescribed without relief; anodynes, blisters, cathartics and the whole routine of an antiphlogistic course had been severally tried, and all proved equally inefficient. The disease would generally take its own course during these severe paroxysms, and gradually exhaust itself. The constitution was very much impaired by these frequent and violent attacks, and the faculties of the mind were more or less compromised. The variety of means which had been resorted to, together with the character of the medical gentlemen who had prescribed for the case, had induced the patient, who was an intelligent man, to believe that nothing could be done for his relief; and it was with little faith that he heard my suggestions. I ventured modestly to assure him that, so far as his disease was of neuralgic origin, the Aconite would cure it. He yielded to my solicitation and commenced the use of Fleming's Tincture, taking ten drops three times a day; gradually increasing the dose if it should be found necessary to 20 drops. I had the satisfaction of seeing the gentleman at my house some four weeks from this time, and received the assurance that he had not been so well for several years. He was looking hale and robust, and was free from the neuralgia.

I am in the habit of prescribing it in the low agues so common in our Western country in paludal districts, and in such cases its efficiency is remarkably increased by being given in combination with Quinine. As many of your readers may be unable to find the formula for its preparation, I will transcribe it as given in Ranking:

"Take of root of A. Napellus, carefully dried and finely powdered, 16 ozs. Troy, rectified Spirit 16 fluid ounces; macerate for four days, then pack into a percolater; add rectified Spirit until 24 ounces of Tincture are attained."

It is beautifully transparent, of the color of sherry, and the taste is slightly bitter. The average dose is five minims (about 10 drops) three times daily, though I have given as many as twenty drops three times a day in a case of severe sciatica with no unpleasant results.

*Feb. 16th,* 1852.

